# Negative Correlation Between Functional Connectivity and Small-Worldness in the Alpha Frequency Band of a Healthy Brain

**DOI:** 10.3389/fphys.2020.00910

**Published:** 2020-08-12

**Authors:** Laura Päeske, Hiie Hinrikus, Jaanus Lass, Jaan Raik, Maie Bachmann

**Affiliations:** ^1^Centre for Biomedical Engineering, Department of Health Technologies, School of Information Technologies, Tallinn University of Technology, Tallinn, Estonia; ^2^Department of Computer Systems, School of Information Technologies, Tallinn University of Technology, Tallinn, Estonia

**Keywords:** electroencephalography, functional connectivity, small-world organization, network analysis, alpha frequency, coherence, imaginary part of coherency, synchronization likelihood

## Abstract

The aim of the study was to analyze the relationship between resting state electroencephalographic (EEG) alpha functional connectivity (FC) and small-world organization. For that purpose, Pearson correlation was calculated between FC and small-worldness (SW). Three undirected FC measures were used: magnitude-squared coherence (MSC), imaginary part of coherency (ICOH), and synchronization likelihood (SL). As a result, statistically significant negative correlation occurred between FC and SW for all three FC measures. Small-worldness of MSC and SL were mostly above 1, but lower than 1 for ICOH, suggesting that functional EEG networks did not have small-world properties. Based on the results of the current study, we suggest that decreased alpha small-world organization is compensated with increased connectivity of alpha oscillations in a healthy brain.

## Introduction

Functional connectivity (FC) is highly important in physiology at various levels: from molecules to organs and physiological networks are not only of wide scientific interest, but also have high impact in medicine (Ivanov et al., 2016; Lin et al., 2016; Moorman et al., 2016). Functional connectivity is crucial also in brain physiology (Lynn and Bassett, 2019). Significant work has been done to show that neural network architecture can be adaptively reconfigured between different states of the subjects (Bassett et al., 2006; Liu et al., 2015a; Lin et al., 2020) and associate network topology to physiologic states (Bashan et al., 2012; Bartsch and Ivanov, 2014; Ivanov and Bartsch, 2014; Bartsch et al., 2015; Liu et al., 2015b).

Functional connectivity and complex network analysis have been the most widely used types of brain network analysis by providing the tools to analyze the brain as a network of interacting regions, while maintaining computational simplicity. Complex network analysis is based on classical graph theoretical analysis, but focuses on analyzing complex real-life networks (Rubinov and Sporns, 2010). Real-life neural networks are represented graphically, using electroencephalographic (EEG) channels as nodes and FC as edges between nodes. Graphs are constructed by removing edges with lowest values. Small-world organization is one of the most frequently analyzed topological properties of functional neural networks. A network is compared to random networks and in order to have small-world properties, the network should be more clustered than a random network, but have similar characteristic path length (Watts and Strogatz, 1998; Albert and Barabási, 2002; Rubinov and Sporns, 2010; Bassett and Bullmore, 2017). In that case, functional integration and functional segregation are simultaneously high. A measure of small-worldness (SW) has been proposed to assess small-world properties of a network (Humphries and Gurney, 2008). Since then, studying small-world properties of functional brain networks has been widely used.

Changes in EEG resting state FC and small-world structure are often used for statistical analysis between two populations, generally with the aim to compare patient and control groups. Previous studies have found results in all frequency bands, but often inconsistencies between studies occur. Therefore, we will focus on frequency bands, where the most frequent and consistent results were reported. Major depressive disorder (MDD) is mostly characterized by increased FC (Fingelkurts et al., 2007; Leuchter et al., 2012; Olbrich et al., 2014; Li et al., 2017) and more random network structure (Li et al., 2017; Zhang et al., 2018; Sun et al., 2019) in theta and alpha frequency bands. However, few studies have also found a decrease in alpha FC (Shim et al., 2018; Zhang et al., 2018). Alzheimer’s disease (AD) has been consistently characterized by decreased FC in alpha frequency band (Koenig et al., 2005; Wang et al., 2014; Babiloni et al., 2016). Furthermore, SW of AD subjects has been found to decrease in theta frequency band (Wang et al., 2014; Vecchio et al., 2017), and AD is characterized by more random network structure in alpha frequency band (Wang et al., 2014; Babiloni et al., 2016). In schizophrenia, most consistent FC alteration has also been the decrease of FC in alpha frequency band (Jalili and Knyazeva, 2011; Di Lorenzo et al., 2015; Maran et al., 2016). Furthermore, schizophrenia has also been associated with decreased SW in alpha, beta, and gamma frequency bands (Micheloyannis et al., 2006) and more random network architecture (Rubinov et al., 2009).

Although alterations in FC and small-world organization have been studied for diseased brain (see above), the relationship between FC and SW is unclear for healthy subjects. We have previously shown that adding graph theoretical measures to features of FC did not improve classification accuracy when classifying MDD and healthy subjects (Orgo et al., 2017). Therefore, a fundamental relationship between FC and graph theory measures can be expected and a disruption in that relationship is likely related to different mental disorders. However, only a few studies have analyzed the relationship between different graph theory measures. Lynall et al. (2010) reported a positive correlation between functional magnetic resonance imaging (fMRI) FC and SW, together with several correlations between different graph theoretical measures. However, healthy and schizophrenic subjects were analyzed together and the group contained of a small number of subjects (15 healthy and 12 schizophrenic subjects). To the best of our knowledge, the relationship between graph theory measures for EEG data has not been analyzed before.

FC has recently been shown to be a complex spatiotemporal phenomenon (Racz et al., 2018), but in the current study we apply widely used static approach of FC to construct functional networks. To ensure more reliable results, we calculate three frequently used FC measures: magnitude-squared coherence (MSC), imaginary part of coherency (ICOH), and synchronization likelihood (SL). These measures were chosen to take different EEG properties into account. Firstly, SL is calculated in time domain, while MSC and ICOH are calculated in frequency domain. Secondly, measures of FC can be divided into linear and nonlinear measures. On the one hand, EEG nonlinear time series analysis is based on the nonlinear nature of neural processes. Previous studies have reported strong nonlinear interdependences in EEG signals (Rubinov et al., 2009) and nonlinear metrics can detect nonlinear interdependencies between EEG signals that linear measures cannot. On the other hand, nonlinear measures are computationally expensive and susceptible to noise (Netoff et al., 2006). Linear measures are more robust and can perform as well as nonlinear measures in some cases (Bastos and Schoffelen, 2016; Bachmann et al., 2018). Therefore, a combination of linear and nonlinear measures should provide the most information. In the current study, SL can capture both linear and nonlinear interdependencies between signals. We have previously shown with surrogate data method that SL can detect nonlinearity in 9% of EEG segments, which cannot be detected with linear methods (Päeske et al., 2018). Therefore, SL may provide additional information to other connectivity measures. Thirdly, several FC measures such as MSC are strongly influenced by volume conduction (Bastos and Schoffelen, 2016). One solution to avoid spurious results from volume conduction would be to apply inverse method to the scalp EEG signals and then calculate FC between obtained source signals. The problem with this approach is that perfect inverse method cannot exist (Sarvas, 1987) and therefore accurate FC estimation is not guaranteed. Other option is to use FC measures that are less sensitive to volume conduction, for example ICOH (Christodoulakis et al., 2015; Bastos and Schoffelen, 2016). Imaginary part of coherency measures only phase-shifted relationship between time series, therefore minimizing connectivity between information from the same sources. At the same time, true interactions at zero-phase are also lost and for a more complete understanding, these measures can be calculated complementary to other measures.

In the current study, we analyze the relationship between alpha FC and SW in the resting state for healthy subjects. We use only alpha frequency band, because most of the alterations in FC or SW have been previously found in the alpha frequency band for MDD, AD, and schizophrenia. Furthermore, EEG alpha frequency has an important role in cognitive, sensorimotor, psycho-emotional and physiological processes (Bazanova and Vernon, 2014). It is important to note that although graphs are constructed by thresholding FC values, small-world graph theory measures are normalized. Therefore, mathematically, there is no correlation between FC and normalized graph theory measures for random graphs. If a correlation between FC and SW occurs for a physiological network, but not for a random network, the origin of the correlation is also expected to be physiological. We will also construct random graphs for reference, using Erdös-Rényi model, to ensure that our results could not be derived mathematically.

## Materials and Methods

### Subjects

The subjects were chosen for the experiment according to the following criteria: no epilepsy, no usage of psychotropic medication one month prior to the experiment, no usage of narcotics three months prior to the experiment, no history of head injury or concussion, and no psychiatric disorders at the time of the experiment. Following these criteria, the study was carried out on a group of 80 healthy volunteers from ages 19 to 75, with the mean age of 37 ± 15 years. Out of all subjects, 38 were female and 42 were male. The subjects were asked to abstain from alcohol 24 h and from coffee two hours prior to the EEG recording.

The study was conducted in accordance with the Declaration of Helsinki and was approved by the Tallinn Medical Research Ethics Committee. Informed consent was obtained from each subject before participating in the study.

### Data Recordings

Electroencephalographic signals were recorded using Neuroscan Synamps2 acquisition system (Compumedics, NC, United States) from 30 electrodes (Fp1, Fp2, F7, F3, Fz, F4, F8, FT7, FC3, FCz, FC4, FT8, T7, C3, Cz, C4, T8, TP7, CP3, CPz, CP4, TP8, P7, P3, Pz, P4, P8, O1, Oz, O2). Electrodes were positioned according to the extended international 10–20 system with linked mastoids as reference. In addition, horizontal and vertical electrooculograms were recorded to monitor eye movements. To ensure good conductivity between the skin and electrodes, electrode impedances were kept below 10 kΩ.

The data were sampled at 1000 Hz. The resting state EEG was recorded for six minutes, during which the subjects were lying in a relaxed position with their eyes closed. The room of the recordings was electrically shielded and dimly lit. In addition, earplugs were used to minimize any disturbances.

### Preprocessing

The data were analyzed using MATLAB (The Mathworks, Inc.). Butterworth filter was used to filter signals into alpha (8-12 Hz) frequency band. Sampling frequency was reduced to 200 Hz to reduce the computation time of FC measures and the data were divided into 20.48-s (4096 sample) segments. All segments were inspected by a studied technician and segments with ocular, muscular or other artifacts were removed. For each subject, first 10 artifact-free segments were used for further analysis.

Signals were re-referenced according to the reference electrode standardization technique (REST) (Yao, 2001), which approximately re-references scalp EEG signals to a reference point at infinity using an equivalent source model. REST has been shown to be the best reference montage to recover the real EEG FC network configuration (Qin et al., 2010; Huang et al., 2017).

### FC Analysis

Three non-directed measures of FC were calculated in the current study: MSC, ICOH, and SL. An example of EEG signals in alpha frequency band for different levels of FC is shown in the Supplementary Material. FC measures were calculated between all channels, obtaining connectivity matrices for each subject. Median values of MSC, ICOH, and SL were obtained over segments in time.

#### Magnitude-Squared Coherence

Coherency estimates linear relationship between two signals at each frequency *f*. When time series from channels *i* and *j* are *x*_*i*_(*t*) and *x*_*j*_(*t*) and their Fourier transforms are *X*_*i*_(*f*) and *X*_*j*_(*f*), then the cross-spectrum between *X*_*i*_(*f*) and *X*_*j*_(*f*) is $S_{i\,j}\equiv \left\langle X_{i}\,\left(f\right)\,X_{j}^{*}\,\left(f\right)\right\rangle$, where * indicates complex conjugation and ⟨⟩ expectation value. Coherency is calculated as:

(1) $$C_{i\,j}\,\left(f\right)\equiv \frac{S_{i\,j}\,\left(f\right)}{{\left(S_{i\,i}\,\left(f\right)\,S_{j\,j}\,\left(f\right)\right)}^{1/2}},$$

where *S*_*ii*_(*f*) is the power spectrum of *X*_*i*_(*f*) and *S*_*jj*_(*f*) is the power spectrum of *X*_*j*_(*f*). Coherence is the absolute value of coherency:

(2) $$C\,O\,H_{i\,j}\,\left(f\right)\equiv \left|C_{i\,j}\,\left(f\right)\right|=\frac{\left|S_{i\,j}\,\left(f\right)\right|}{{\left(S_{i\,i}\,\left(f\right)\,S_{j\,j}\,\left(f\right)\right)}^{1/2}}$$

In the current study, the MSC (Kay, 1988) was used as a frequently used measure of FC:

(3) $$M\,S\,C_{i\,j}\,\left(f\right)=C\,O\,H_{i\,j}^{2}\,\left(f\right)\equiv \frac{{\left|S_{i\,j}\,\left(f\right)\right|}^{2}}{\left(S_{i\,i}\,\left(f\right)\,S_{j\,j}\,\left(f\right)\right)}$$

Symmetric Hann window with a window length of 512 samples and 50% overlap was used to calculate Fourier transform. MSC was found by averaging *MSC*_*ij*_(*f*) values within the alpha frequency band.

#### Imaginary Part of Coherency

It is often argued not to use MSC as it is strongly influenced my volume conduction. Therefore, ICOH (Nolte et al., 2004) was also used in current study as a secondary measure of FC, which is calculated as an imaginary part of coherency:

(4) $$i\,C\,O\,H_{i\,j}\,\left(f\right)\equiv I\,m\,a\,g\,\left(C_{i\,j}\,\left(f\right)\right)=\frac{I\,m\,a\,g\,\left(S_{i\,j}\,\left(f\right)\right)}{{\left(S_{i\,i}\,\left(f\right)\,S_{j\,j}\,\left(f\right)\right)}^{1/2}}$$

Imaginary part of coherency was found by averaging *iCOH*_*ij*_(*f*) values within the alpha frequency band. Imaginary part of coherency removes zero-phase interactions between time series *x*_*i*_(*t*) and *x*_*j*_(*t*), therefore minimizing the effects of volume conduction.

#### Synchronization Likelihood

Synchronization likelihood (Stam and Van Dijk, 2002) describes dynamical interdependencies between simultaneously recorded signals. The definition and calculation of SL is provided by Stam and Van Dijk (2002). Briefly, time series are reconstructed in state space and the recurrences of states are detected from time-delay embedding vectors. Synchronization likelihood is the likelihood of these recurrences being simultaneous. The parameters for SL were calculated from sampling frequency, highest frequency and lowest frequency using suggestions by Montez et al. (2006). Therefore, the following parameters were used: the embedding lag *L* = 6, the embedding dimension *m* = 6, the number of recurrences nrec = 10, the fraction of recurrences pref = 0.01, window W1 = 50 and window W2 = 1049. Such selection of the parameters ensures that the state vector is long enough to sample the slowest oscillations and at the same time signal is sampled at sufficiently short intervals to take fastest oscillations into account.

### Graph Theory Analysis

A connectivity matrix can be analyzed as a graph consisting of nodes (EEG channels) and edges between the nodes (FC between EEG channels). To obtain a graph, a threshold is applied on FC values: an edge exists only if the value of FC is higher than the threshold. In the current study, the sparsity of each graph was maintained by applying a different threshold to each graph. For example, network density of 40% means that 60% of all connections were removed from each graph. This ensures that differences between graph theory metrics are due to differences in graph topologies, rather than connectivity strengths. As currently there is no optimal network density used in the literature, a range of network densities are used. In the current study, the network densities from 10 to 50% with a step of 5% were used. These are one of the commonly analyzed densities, ensuring that the network is sparse enough to show small-world properties and at the same time is still fully connected (Bullmore and Bassett, 2011; Sun et al., 2019). Obtained graphs were binarized: edge values were 0 or 1, depending on whether there was a connection between two nodes or not. In other words, unweighted graphs were used in the current study. As non-directed FC measures were used, edges did not have a direction.

Brain Connectivity Toolbox (Rubinov and Sporns, 2010) was used to calculate graph theoretical measures in MATLAB. Graph theory measures calculated in the current study describe small-world properties of a network and are therefore also called small-world measures. Clustering coefficient (*C*) describes functional segregation, characterizing brain’s ability to process information within interconnected clusters. Clustering coefficient for a given node equals with the fraction of node’s nearest neighbors that are also directly connected to each other (Watts and Strogatz, 1998). Characteristic path length (*L*) is a measure of functional integration, characterizing brain’s ability to combine information from distributed areas. Shortest path length is the smallest number of edges between two nodes. Characteristic path length is the average shortest path length of the graph (Watts and Strogatz, 1998). High functional integration is described with small *L*. A network has small-world properties if it is more clustered than a random network, but has similar *L* (Rubinov and Sporns, 2010). Small-worldness quantifies these properties and is calculated from *C* and *L* (Humphries and Gurney, 2008):

(5) $$S\,W=\frac{C_{n\,o\,r\,m}}{L_{n\,o\,r\,m}}=\frac{C/C_{r\,a\,n\,d}}{L/L_{r\,a\,n\,d}},$$

where *C*_*rand*_ is the clustering coefficient and *L*_*rand*_ is the characteristic path length of an equivalent random network. A network has small-world properties if SW > 1 (Wang et al., 2014). Random networks for normalization were generated according to the method of Maslov and Sneppen (Maslov and Sneppen, 2002; Rubinov and Sporns, 2010) by reshuffling the topology and maintaining the degree distribution of original networks.

Small-worldness was calculated for all three FC measures. For reference, random graphs were generated using Erdös–Rényi model – for the fixed number of nodes, the existence of each potential edge is determined by a probability *p*. To differentiate between graph theoretical measures calculated from different FC measures, FC measures are marked with a superscript. For example SW^MSC^ denotes small-worldness, calculated from a MSC graph.

### Statistical Comparisons

For each subject, the mean values of FC and SW were calculated over all channels. The values of SW were statistically compared using Wilcoxon’s ranksum test and the correlations between mean FC and SW were calculated using Pearson correlation coefficient (*r*). The confidence level of *p* < 0.05 was used. *p*-Value was adjusted according to the number of statistical tests using Bonferroni correction to address the problem of multiple comparisons. As three different measures and nine different graph densities were used, the number of statistical tests was 27 and *p*-value was adjusted to *p* < 0.05/27 = 0.0019. The correlations were considered statistically significant if |*r*| > 0.34, corresponding to the adjusted *p*-value *p* < 0.0019 and sample size of 80 subjects. If the absolute value of obtained correlation was higher than 0.34, the correlation could not have emerged randomly.

## Results

**FIGURE 1:**
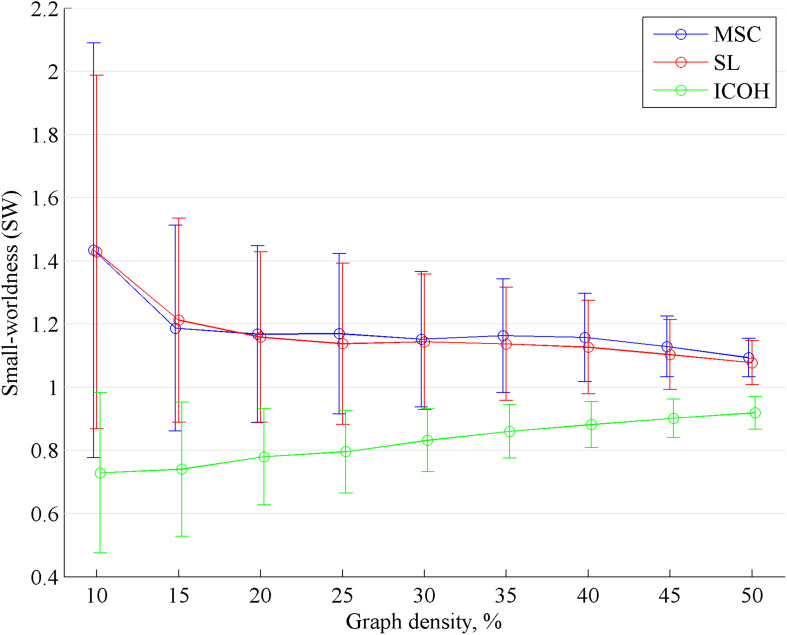
Small-worldness (SW) depending on graph density. The mean and standard deviation values are shown for SW, calculated from MSC, SL and ICOH. SW^ICOH^ is significantly lower than SW^MSC^ and SW^SL^.

First, we statistically compared the values of SW calculated from different FC measures (Figure 1). Bonferroni correction for 27 statistical tests was applied. Small-worldness calculated from ICOH was significantly lower than SW calculated from MSC and SL for all graph densities analyzed in the current study. For MSC and SL, SW was mostly above 1 or close to 1, indicating these networks have better or similar small-world properties compared to a random network. For ICOH, most values of SW were below 1, indicating these networks have less small-world properties compared to a random network.

**FIGURE 2:**
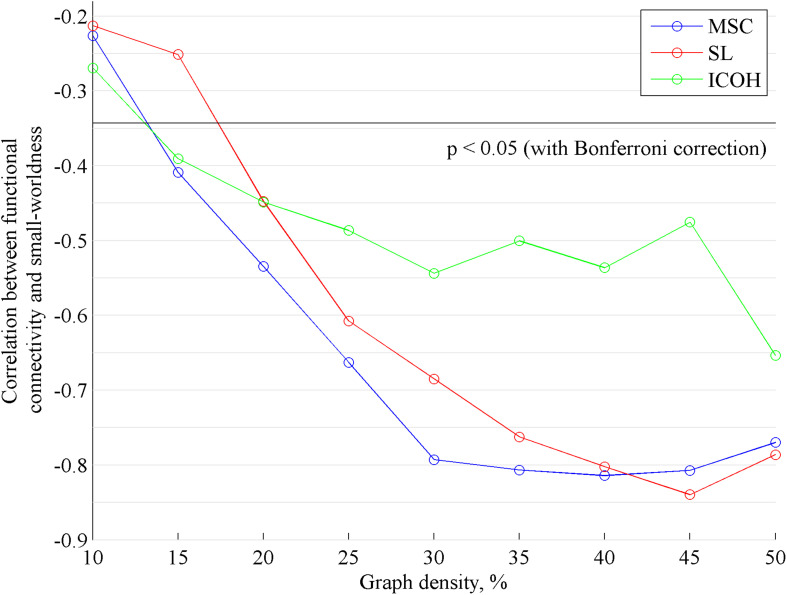
The values of Pearson correlation coefficients (*r*) between measures of functional connectivity (MSC, SL, and ICOH) and small-worldness calculated from these measures (SW^MSC^, SW^SL^, and SW^ICOH^) for different graph densities. Black horizontal line corresponds to correlation -0.34. Correlations below this line are statistically significant with confidence level of 0.05 (*p*-value is adjusted according to Bonferroni correction to *p* < 0.0019).

**FIGURE 3:**
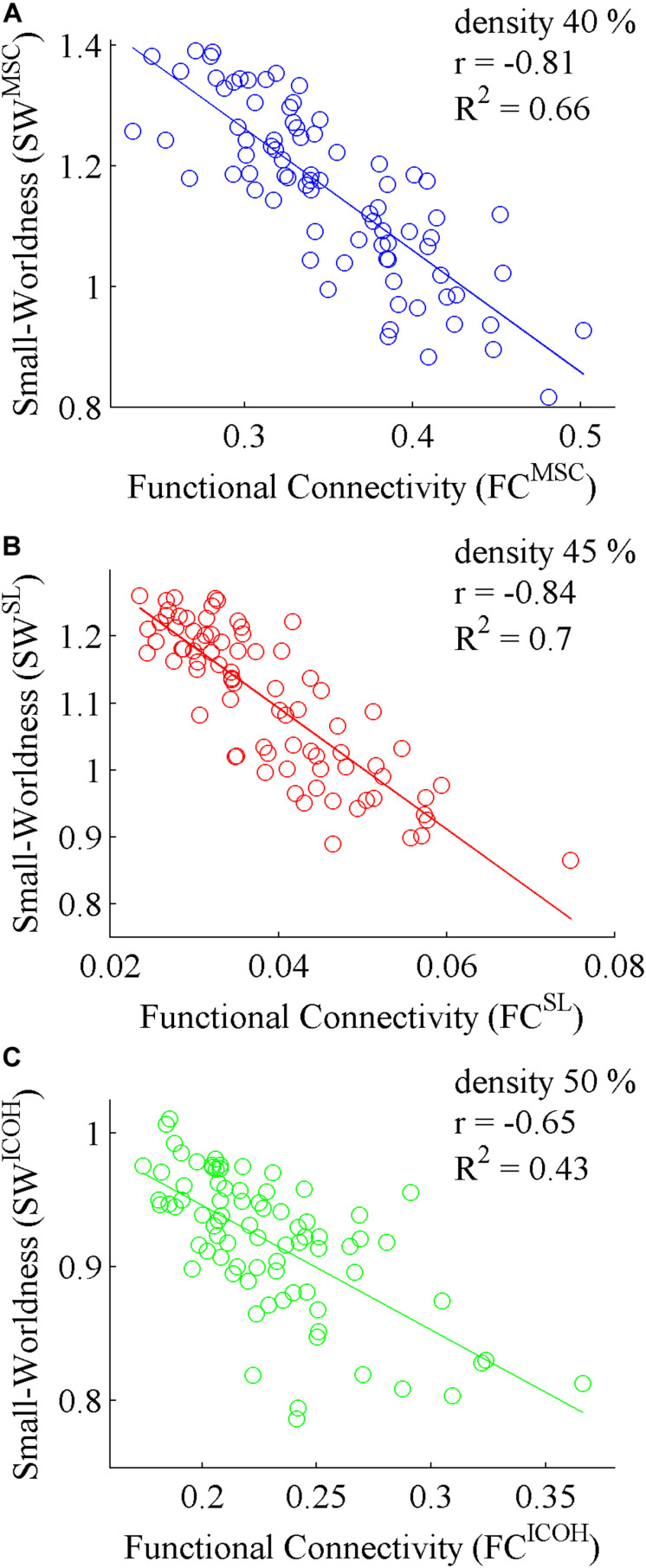
Correlation between FC and SW for **(A)** MSC, **(B)** SL, and **(C)** ICOH. Pearson correlation coefficient (*r*) is shown in the upper right corner.

Secondly, Pearson correlation coefficient was calculated between SW and FC for all measures of FC (Figure 2). There was a statistically significant negative correlation between FC and SW for all measures of FC. For MSC and ICOH, correlations were statistically significant for graph densities 15 … 50% and for SL 20 … 50%. The highest correlations are plotted on Figure 3. The highest correlation for MSC was for graph density 40% (Figure 3A), for SL 45% (Figure 3B) and for ICOH 50% (Figure 3C). Pearson correlation coefficient was also found between SW and averaged edge values of random graphs. As expected, correlation for random graphs was not statistically significant for any of the analyzed graph densities.

## Discussion

As a result of the study, we found a negative correlation between EEG alpha FC and SW. The correlation occurred for all three measures of FC calculated in the current study. For MSC and ICOH, correlations were statistically significant for graph densities 15 … 50% and for SL 20 … 50%. Based on the results of the current study, we suggest a hypothesis that decreased alpha small-world organization is compensated with increased connectivity of alpha oscillations in a healthy brain. Furthermore, a correlation may indicate that a certain efficiency is maintained in the brain by balancing between alpha FC and SW: as one increases, the other decreases.

Results found in the current study may be associated with default mode network (DMN; Jann et al., 2010; Liu et al., 2017). The DMN has been the most studied of resting state networks, largely because it deactivates during demanding tasks. Furthermore, areas involved in DMN have high activity during resting state, observed with fMRI BOLD signal, and high connectivity (Hagmann et al., 2008). A recent study used high-density EEG to detect large-scale networks (Liu et al., 2017). The authors spatially overlapped obtained EEG networks with fMRI networks and found that although each resting state brain network is associated with oscillations of different frequency bands, DMN can be fully reconstructed using alpha frequency band. In the current study, alpha frequency band was also used and therefore association between the results in the current study and DMN are plausible.

Previous studies have mostly found that alpha FC is increased in MDD (Fingelkurts et al., 2007; Leuchter et al., 2012; Olbrich et al., 2014). Although changes in alpha SW in MDD are unclear, some studies have found that small-world measures of alpha EEG were decreased for subjects with MDD (Zhang et al., 2018; Sun et al., 2019). Therefore, in MDD, the relationship between FC and SW found in the current study is probably not disrupted. Fingelkurts et al. (2007) suggested that FC between short-range connections in the left hemisphere and long-range connections in the right hemisphere of subjects with MDD was increased to compensate insufficient semantic integration. However, according to the hypothesis suggested in the current study, the compensational mechanism proposed by Fingelkurts et al. (2007) may be inherent to healthy subjects as well. A compensational mechanism could be a fundamental characteristic to brain functioning. According to that theory, another possible explanation to the increase in FC for MDD is the decrease in SW, which in turn leads to an increase in FC.

Alzheimer’s disease in alpha frequency band is characterized by decreased FC (Koenig et al., 2005; Wang et al., 2014; Babiloni et al., 2016), but also decreased small-world measures (Wang et al., 2014; Babiloni et al., 2016). Therefore, compensating low small-world architecture with increased FC may be disrupted in AD.

Similarly to AD, schizophrenia in alpha frequency band has also been previously described with decreased FC (Jalili and Knyazeva, 2011; Di Lorenzo et al., 2015; Maran et al., 2016) and small-world measures (Micheloyannis et al., 2006; Rubinov et al., 2009). Schizophrenia is often described with “dysconnectivity syndrome” – impaired functional integration between and within brain areas. Considering the results of the current study, it could be presumed that “dysconnectivity syndrome” is expressed by disrupted compensational mechanism in schizophrenia.

We statistically compared the values of SW calculated from different FC measures. Small-worldness calculated from ICOH (SW^ICOH^) was significantly lower compared to SW^MSC^ and SW^SL^. As ICOH measures only phase-shifted relationship between time series, this result shows that MSC and SL capture a lot of information from zero-phase interactions. A lot of these interactions are due to volume conduction. Previous studies have shown that volume conduction falsely increases values of SW (Kuś et al., 2004). The same effect could also be observed in the current study, where SW^MSC^ and SW^SL^ were significantly higher compared to SW^ICOH^. Nevertheless, the correlation between FC and SW found in the current study cannot be caused by volume conduction, because in addition to MSC and SL, the correlation was also found with ICOH, which minimizes the effects of volume conduction.

Magnitude-squared coherence is a linear measure that is calculated in a frequency domain and SL is a nonlinear measure that is calculated in a time domain. Although these measures are fundamentally different, there were no statistically significant differences between SW^MSC^ and SW^SL^. This result shows that for robust network analysis applications, MSC can be selected instead of SL, because MSC is easier and faster to compute.

In the current study, SW^MSC^ and SW^SL^ were mostly slightly higher than 1 (Figure 1), indicating these networks have better or similar small-world properties compared to the random networks generated from original networks. However, SW^ICOH^ was mostly below 1 (Figure 1), indicating these networks have less small-world properties compared to a random network. These results are in line with previous studies: SW has been found to be above 1 for FC measures that are more influenced by volume conduction (Micheloyannis et al., 2006; Wang et al., 2014; Zhang et al., 2018) and slightly below 1 for measures that are less influenced by volume conduction (Hou et al., 2018; Zheng et al., 2018). Previous studies have found that EEG functional networks are small-world networks, but the current study shows that these results may be influenced by volume conduction, since functional ICOH networks in the current study did not show small-world properties during eyes-closed resting state.

Most studies that compare two groups of subjects, obtain values above 1 for SW. Since those metrics are obtained by comparing original networks to random networks, decrease in those values is generally interpreted as a more random network structure (Rubinov et al., 2009; Zhang et al., 2018; Sun et al., 2019). In the current study we showed that although decrease in SW^MSC^ and SW^SL^ can be interpreted as a more random network structure (Figures 3A,B), decrease in SW^ICOH^ resulted in a less random network structure (Figure 3C). Therefore, the decrease in SW does not necessarily interpret into a more random network structure, although such result can be concluded mathematically in case of certain measures. These results strengthen the argument to calculate ICOH in addition to MSC or SL.

The negative correlation obtained in the current study increased between graph densities 10 … 25% and was more stable for graph densities 30 … 50% (Figure 2). As mean FC was constant for all graph densities, this result could be more influenced by the dependence of SW on graph density. Still, one has to take into account that the dependence of SW on graph density differs for each individual network. Generally, denser networks naturally have smaller values of SW (Bassett and Bullmore, 2017). However, the same conclusion did not apply to the results of ICOH in the current study (Figure 1). To address the limitation of SW depending on the graph density, the small-world propensity (SWP) was introduced by Muldoon et al. (2016). However, in the current study, we chose a more common approach to calculate SW for a range of graph densities (Figure 2) to investigate the correlation between FC and SW depending on the graph density.

In the current study, functional networks of healthy subjects in resting state was analyzed. Further studies could also investigate the relationship between FC and SW in subjects with MDD, AD, and schizophrenia. Based on the network analysis in studies by other authors, the relationship between FC and SW found in the current study may be disrupted in AD and schizophrenia, but not in MDD.

Previous studies have shown that different physiologic states can be described with different network structure (Bartsch et al., 2015) and FC (Lin et al., 2016) within organ systems, indicating an association between network topology, FC, and physiologic function. In the more focused perspectives of the brain, the hypothesis of a compensatory mechanism between FC and SW suggested in the current study seems to be consistent with these findings in that in healthy subjects FC and SW underlying different physiologic states may well alter in an interrelated manner. This concept should be made subject of further research within a broader framework incorporating functional integration and segregation, too.

## Conclusion

To the best of our knowledge, current study is the first to analyze the relationship between resting state EEG FC and SW. We report a negative correlation between FC and small-world organization in alpha frequency band for healthy subjects. We interpret these results as the manifestation of a compensational mechanism of the healthy brain, where lower small-world organization is compensated by higher connectivity strength. The finding is expected to be useful in the differentiation of mental and neurological disorders.

## Data Availability Statement

All datasets presented in this study are included in the article/Supplementary Material.

## Ethics Statement

The studies involving human participants were reviewed and approved by Tallinn Medical Research Ethics Committee, Estonia. The patients/participants provided their written informed consent to participate in this study.

## Author Contributions

LP designed the study and processed the data. LP and MB conducted the EEG recordings. LP, MB, and HH analyzed and interpreted the results and wrote the manuscript. JL and JR contributed to the discussion of results and writing the manuscript. All authors revised and approved the final manuscript.

## Conflict of Interest

The authors declare that the research was conducted in the absence of any commercial or financial relationships that could be construed as a potential conflict of interest.
