# Supplementary material for: Negative Correlation Between Functional Connectivity and Small-Worldness in the Alpha Frequency Band of a Healthy Brain
Source: Front Physiol. 2020 Aug 12;11:910. doi: 10.3389/fphys.2020.00910 (PMC7437013; doi:10.3389/fphys.2020.00910)
Supplement: Supplementary file 1 [file Image_1.pdf]

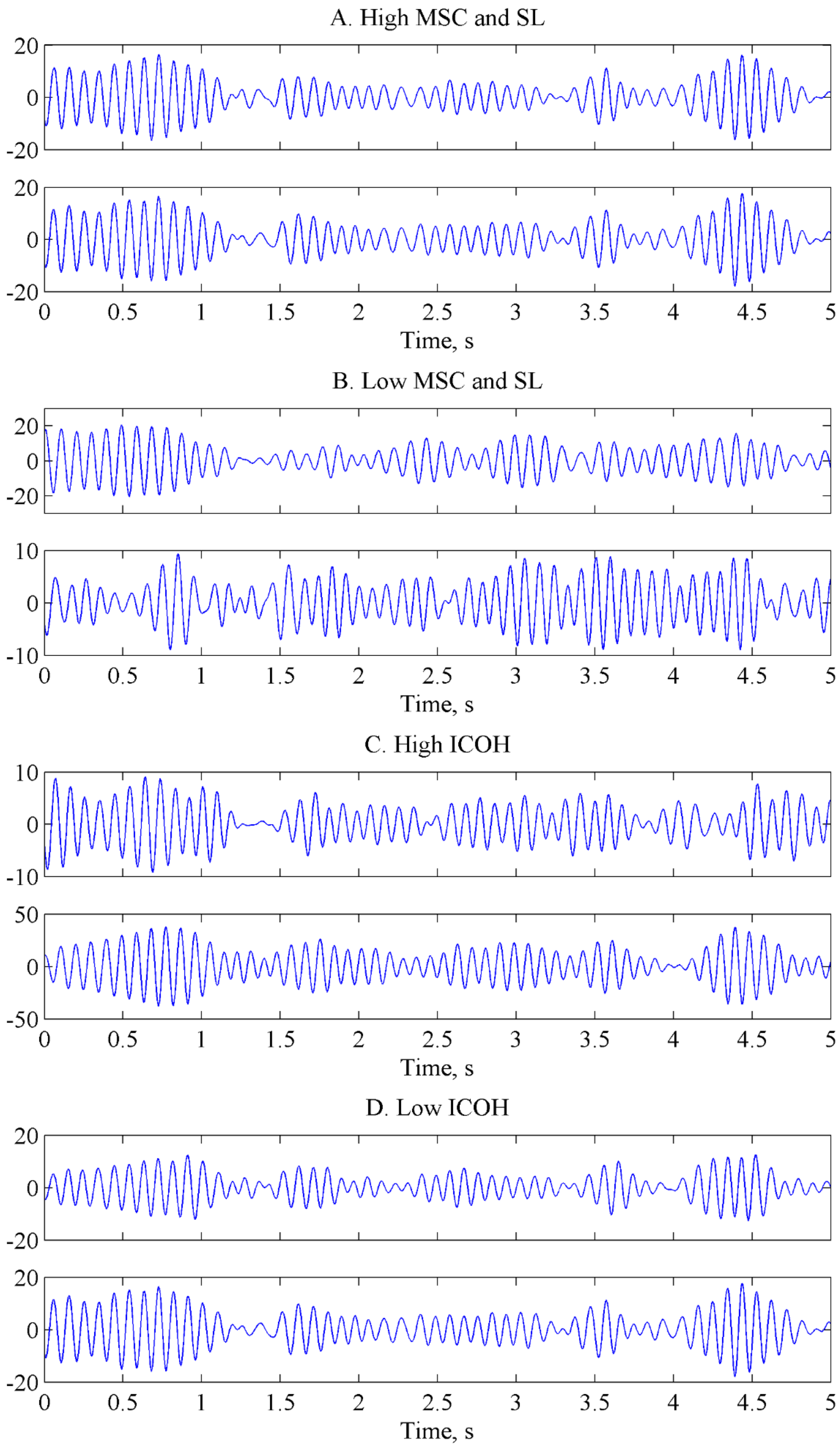

Supplementary Figure 1. An example of 5-second EEG signals from different channels of one subject. (A) Signals from channels FC4 and F4. MSC and SL between these signals is high: 0.95 for MSC and 0.52 for SL; (B) Signals from channels P3 and P8. MSC and SL between these signals is low: 0.14 for MSC and 0.02 for SL; (C) Signals from channels OZ and CP4. ICOH between these signals is high: 0.42; (D) Signals from channels FT8 and F4. ICOH between these signals is low: 0.14.
